# LRP1: a novel receptor for the transmission of pathological α-Synuclein

**DOI:** 10.1186/s13024-022-00582-4

**Published:** 2022-11-26

**Authors:** Crystal Pan, Chao Peng

**Affiliations:** Department of Neurology, David Geffen School of Medicine, University of California - Los Angeles, 90095 Los Angeles, CA USA

**Keywords:** LRP1, α-Synuclein, Parkinson’s disease, Receptor mediated endocytosis

During the past decade, mountains of evidence support that the transmission of pathological proteins is a shared mechanism for the progression of various neurodegenerative diseases [1]. α-Synuclein (α-syn) is the pathological protein for a group of neurodegenerative diseases collectively known as α-synucleinopathies, including Parkinson’s disease (PD), dementia with Lewy body (DLB), multiple system atrophy and ~ 50% of Alzheimer’s disease (AD). Blocking the transmission of pathological α-syn represents a promising therapeutic strategy to slow down or reverse the progression of these diseases. Therefore, molecular mechanisms that mediate the transmission of pathological α-syn are attractive drug targets. However, despite extensive study, the molecular mechanisms underlying the transmission of pathological α-syn remains unknown.

Various different mechanisms have been proposed to mediate the transmission of pathological proteins, such as tunneling nanotubes and exosomes [1]. Multiple cell surface receptors have also been reported to bind with pathological proteins and mediate their transmission (Table 1). In 2016, Mao et al. reported that lymphocyte-activation gene 3 (LAG3) receptor mediated the transmission of pathological α-syn in animal and cell models [2]. α-Syn preformed fibrils (PFF) bind directly to the extracellular immunoglobulin-like domains of LAG3. Blocking the interaction between α-syn and LAG3 could reduce the transmission of pathological α-syn. In 2013, Holmes et al. showed in cell models that heparan sulfate proteoglycan (HSPG) mediated macropinocytosis is responsible for the uptake of both pathological α-syn and tau [3]. HSPGs have also been reported to mediate the endocytosis of amyloid-beta (Abeta) [4]. Members of the low-density lipoprotein receptor (LDLR) family have been reported as co-receptors for HSPGs [4, 5]. In 2020, through a functional screen of LDLR receptors, Rauch et al. demonstrated that low-density lipoprotein receptor-related protein 1 (LRP1), is a key receptor that mediated the interneuronal spreading of pathological tau. LRP1 is a member of the LDLR family and expressed in a wide variety of cell types including neurons, astrocytes, microglia, macrophages, fibroblasts, and smooth muscle cells. LRP1 mediates the internalization of physiological tau and pathogenic tau oligomers [6]. LRP1 has also been reported as a co-receptor for HSPG that binds and uptakes Abeta [5]. Moreover, LRP1 has been reported to interact with ApoE, the most significant genetic risk factor for Alzheimer’s disease [7].

**Table 1:** Receptors mediating the transmission of pathological proteins

Receptor	Pathological Proteins
HSPGs	Abeta [5], tau [3], α-syn [3]
LAG3	α-syn [8]
LRP1	Abeta [3], tau [6], α-syn [8]

Given that HSPGs have been reported to mediate the endocytosis of pathological α-syn, LRP1, as a co-receptor for HSPGs, could also play a critical role in the transmission of pathological α-syn. Published in *Molecular Neurodegeneration* on September 2, 2022, Chen and colleagues explored the role of the LRP1 in the transmission of α-syn using a wide variety of different models, including LRP1 knockout human induced pluripotent stem cell (iPSc) derived neurons and neuronal LRP1 deletion mice. Loss of LRP1 significantly reduced the internalization of monomeric α-syn, effectively inhibited the uptake of α-syn oligomers, and mildly inhibited the uptake of α-syn PFFs in vitro, suggesting that LRP1 is a mediator for the uptake of soluble α-syn. Using α-syn with modified lysine residues at the N-terminus of the protein, the authors demonstrated that lysine residues and the N-terminus are critical for the interaction and internalization of α-syn. The authors then established a murine model that discriminates between neurons transduced with the adeno-associated virus expressing human α-syn and neurons that receive human α-syn through spreading. Using this novel model, they demonstrated that the deletion of LRP1 in the neurons suppresses the transmission of human α-syn in mice [8].

Illustrating the role of LRP1 in the transmission of α-syn, particularly monomers and oligomers, provides critical insights into the interneuronal transmission mechanism of α-syn, and highlights LRP1 as a promising drug target to block the spreading of oligomeric α-syn in α-synucleinopathies. The usage of various models in the study also dramatically strengthened the conclusion. There are also some questions that remain to be addressed. The very mild effect of LRP1 deletion on α-syn PFF transmission indicates that LRP1 might mediate only a portion of pathological α-syn transmission. Pathological α-syn has been shown to form different conformations, and it remains unknown whether LRP1 plays similar roles in the transmission of various different pathological α-syn conformations. It is also unknown whether post-translational modifications such as phosphorylation on pathological α-syn would modulate the interaction with LRP1. How the existence of co-pathologies such as pathological Abeta and tau would affect LRP1 mediation of pathological α-syn transmission is also unknown. Finally, only the transmission of α-syn monomer has been tested in vivo. Future studies should further explore how LRP1 deletion affects α-syn oligomer and fibril transmission in vivo.

The observation that LRP1 plays a much more important role in the transmission of α-syn monomers and oligomers than fibrils is interesting. The mechanisms, by which LRP1 distinguishes α-syn oligomers versus fibrils remain unknown. Given that α-syn oligomer is a heterogeneous population and the cutoff between fibrils and oligomers is not clearly defined, it would be interesting to further explore the exact size of α-syn oligomers that can be taken up by LRP1 using size exclusion chromatography. Pathological α-syn in diseased brains has been suggested to be a heterogenous population, so the selective uptake of certain pathological species by LRP1 could contribute to selective vulnerability of different brain regions and patients.

Chen and colleagues provide new insights into α-syn transmission. This study highlights LRP1 as a promising drug target for α-synucleinopathies. It also raises many questions that are worth further studies. The exact mechanism of how LRP1 mediates α-syn transmission as well as the interactions between LRP1 and downstream signaling pathways remains to be fully discovered. Evidence from diseased brains will be needed to further support the role of LRP1 in the etiology of α-synucleinopathies. It would also be interesting to explore the role of other LDLRs in the transmission of pathological α-syn. It is possible that different LDLRs could mediate the transmission of different pathological α-syn species.

## Data Availability

Not applicable.

## Abbreviations

α-syn: α-Synuclein
PD: Parkinson's Disease
DLB: dementia with Lewy body
AD: Alzheimer's disease
LAG3: lymphocyte-activation gene 3
PFF: preformed fibrils
HSPG: heparan sulfate proteoglycan
Abeta: amyloid beta
LDLR: low-density lipoprotein receptor
LRP1: low-density lipoprotein receptor-related protein 1
iPSc: induced pluripotent stem cells
